# Potential Patient-Reported Toxicities With Disulfiram Treatment in Late Disseminated Lyme Disease

**DOI:** 10.3389/fmed.2020.00133

**Published:** 2020-04-20

**Authors:** Alain Trautmann, Hugues Gascan, Raouf Ghozzi

**Affiliations:** ^1^Université de Paris, Institut Cochin, INSERM U1016, CNRS UMR8104, Paris, France; ^2^Institut de Génétique et Développement de Rennes (IGDR), Rennes, France; ^3^Centre Hospitalier de Lannemezan, Lannemezan, France

**Keywords:** lyme disease, disulfiram, benefit, patient-reported adverse drug reactions, risk

## Abstract

Recently, disulfiram has been proposed as a promising treatment for people suffering from persistent symptoms of Lyme Disease. Disulfiram has several distinct molecular targets. The most well-known is alcohol dehydrogenase, a key enzyme for detoxifying the organism after alcohol ingestion. Other targets and modes of action of disulfiram, that may present problematic side effects, are less commonly mentioned. The *French Federation against Tick Borne Diseases* (French acronym, FFMVT), which associates three main Lyme patient organizations, MDs and PhDs, has recently been alerted to severe and persistent toxic events in a patient suffering from a late disseminated form of Lyme Disease following disulfiram intake. FFMVT reacted by launching a national call to examine whether other patients in France following a similar treatment could be identified, and what benefits, or side effects could be reported. The statements of 16 patients taking disulfiram have been collected and are presented here. Thirteen out of 16 patients reported toxic events, and seven out of 16 reported benefits for at least part of their symptoms. Based on the collected observations, it seems too early to promote disulfiram as a promising new treatment until the reasons underlying the reported toxicities have been explored, and the results of a well-conducted double blind clinical trial published. The importance of taking into account patient-reported outcomes in Lyme Disease is underlined by the present study.

## Introduction

Each year, in the USA, about one person out of 1,000 develops Lyme Disease as declared by the general practitioners, which leads to a total of around 300,000 annual cases (1). Similar frequencies have been reported in Europe, in particular in France (2) and in Germany (3). The patients usually take antibiotics for a few weeks, and in most of cases they recover. However, after several months or even years, a fraction of these properly treated patients, will develop a post-treatment Lyme Disease syndrome (PTLDS) linked to pathogens injected by the tick, usually *Borrelia* bacteria, often associated with other bacteria like *Bartonella*, parasites like *Babesia*, or even viruses. Another group of patients develops a late disseminated form of the disease without having noticed any initial event, like the pathognomonic cutaneous *erythema migrans*, rendering more difficult the Lyme Disease diagnosis.

There is no consensus for the optimal treatment of these late forms of disease. The major difficulties in their diagnosis and in their treatment are reflected in the number of different names they have been given: late Lyme Disease, persistent Lyme Disease, chronic Lyme Disease, PTLDS, and in France SPPT for “*sémiologie persistante polymorphe après morsure de tique*” according to the new French government guidelines.

In such a context, there is a desperate need for many people to receive the optimal treatment. Recently, a new treatment has been reported, and many patients are currently trying it despite the fact that the main active compound, disulfiram (DSF), has never been clinically evaluated in the context of a chronic infection, either alone or in combination with antibiotics. In the present work, to try to answer this pressing issue, we have analyzed the scientific literature on DSF and collected patient-reported results in order to inform patients suffering from late forms of Lyme Disease of the potential risks or benefits of DSF treatment.

## Methods

After an alert in October 2019 from a patient presenting severe and persistent symptoms after taking DSF, the FFMVT (*French Federation against Tick Borne Diseases*) decided to launch two actions. One was a thorough analysis of the scientific literature, in order to try to understand the possible causes of such an apparent toxicity. The other one was to collect reported-outcomes from Lyme Disease patients having taken DSF. Three associations of patients, *France Lyme, Lympact, Relais de Lyme*, sent a standardized questionnaire, prepared by the authors, to their members suffering from PTLDS as described, among others, by J. Aucott (4), or from SPPT, the term used in France by *Haute Autorité de Santé* (High Health Authority) (https://www.has-sante.fr/portail/jcms/c_2857558/fr/borreliose-de-lyme-et-autres-maladies-vectorielles-a-tiques) and in a case law of the French Council of State (https://www.conseil-etat.fr/fr/arianeweb/CE/decision/2019-12-04/423060).

Concerned patients who were willing to contribute to this enquiry sent back the appropriate information on their clinical status and disease. Information requested included age, sex, health state, dosage and duration of the DSF treatment, concomitant medications, self-reported health improvements and potential toxicities. The answers were collected over a 2-weeks period, and anonymously transferred from patient associations to the authors of the present paper, before being tabulated and analyzed, as presented in Table 1. Note that the Research Integrity Specialist of Frontiers asked us to omit the gender information, and not to indicate the precise age, to reduce the risk for the patients to be indirectly identifiable.

**Table 1:** Responses of 16 patients to DSF treatment (France, July–November 2019).

**Patient**	**Age**	**Subtype of disease**	**DSF treatment (in mg/day, duration, sequence)**	**Other treatment**	**DSF attributed benefit (patient self-report)**	**DSF attributed side-effects (patient self-report)**
#1	21–25	Late L.D.	250 (1 d/3 for 1 w), (1 d/2 for 1 w), (1 w), 500 (10 d), 250 (3 d)		No	Cranial neuropathy, very strong headaches, anxiety, suicidal thoughts, loss of sleep, hot flushes, disturbed intestinal transit, osteo-articular pain, tremors, tinnitus linked to head movements, hyperacusis. Stopped the treatment, but major side effects persisted after 1 month.
#2	41–45	Late L.D.	250 (5 w), 125 (8 w)	Antibiotic	No	Strong dizziness and nause.
#3	66–70	Late L.D., associated with a suspected lupus	500 (4 w)	Hydroxychloroquine	Clear decrease of fatigue and pain	No
#4	46–50	Late L.D.	250 (2 w), 500 (7 w), 750 (2 w)		Too early	Fatigue, general pains, loss of sleep, tachycardia, paranoid delirium requiring stopping the treatment.
#5	36–40	Late L.D.	62.5 (1 d/3 for 2 w), 62.5 (2 d/3 for 1 w), currently 62.5 (1 w)	Antibiotic, hydroxychloroquine	No	No
#6	46–50	Late L.D.	250 (3 w), a 24 h break, 62.5 (3 w)	Nitazoxa–nide	After a 24 h break, a major effect on fatigue and pain	At 250 mg per day, strong Jarish-Herheimer reactions, with headaches, cranial neuropathy, fatigue, pain.
#7	31–35	Late L.D.	500 (4 w)	Antibiotic	No	No
#8	61–65	Late L.D.	250 (4 w)		No	Important nausea, anorexia, significant weight loss, exacerbation of pain.
#9	45–50	Late L.D.	250 (6 w), 500 (1 w) at night		A decrease of pains was observed	Significant concentration problems when taken during the day. Causes significant disturbances of immediate memory, speech difficulties, headaches and dizziness.
#10	46–50	Late L.D.	125 (2 w)	Antibiotic	No	Flu-like symptoms, increased pain in some joints.
#11	46–50	Late L.D.	250 (4 w)	Antibiotic	Improvement of tone and general health	Dizziness and nausea.
#12	56–60	Late L.D.	250 (1 d/3 for 1 w), 250 (1 d/2 for 1 w), 250 (1 w), 500 (1 w), a 2 w stop		Few days after ending treatment, a real positive effect on the general health with a decline of the majority of symptoms was observed, but 8–10-day later some of them reappeared (leg weakness, joint pain)	Increased pains in the whole body, especially in joints. Important fatigue, flu-like symptoms, low blood tension, eye irritations, abdominal pain, mild nausea.
#13	76–80	Late L.D.	250 (4 w)	Antibiotic	No	Strong dizziness, leading the patient to stop treatment at day 3.
#14	51–55	Late L.D.	From 125 to 500 over 4 w, a 4 w stop, 500 (4 w), 2 w stop, and currently 500		Better sleep. Slight decrease in peripheral nervous system pain (main symptom of this patient)	Fatigue, drowsiness, hot flushes, disturbed intestinal transit.
#15	51–55	Late L.D.	500? (4 w)		No	Major concentration difficulties, strong dizzines, and strong headaches. Important fatigue requiring to lie down.
#16	31–35	Late L.D.	250 (currently under treatment since 3 w)		Improved concentration et cognition. And decrease in headaches and fatigue	Two important Jarish-Herxheimer reactions.

*DSF, Disulfiram; L.D., Lyme Disease; w, week; d, day*.

This enquiry allowed us to rapidly collect the appropriate information for evaluating whether or not reported severe adverse events in a first patient were exceptional or not. However, no definite conclusion can be drawn under such conditions, taking into account the sample size, the different doses of DSF used, and different combinations of concomitant medications used.

## Results

The first part of this section will present potential reasons why toxicity is expected in patients taking DSF, and not exclusively following alcohol ingestion. The second part will concern the analysis of 16 patient-reported outcomes collected in November 2019 in France.

### DSF, an ALDH Inhibitor

DSF has been clinically used for nearly 70 years, essentially for treating alcohol dependence. DSF inhibits an enzyme that is required for full alcohol degradation, preventing the detoxification that should follow alcohol drinking. This leads to severe nausea and discomfort in DSF-treated patients when they drink alcohol. This induced association between alcohol and severe discomfort is the basis of DSF use for the treatment of alcohol-dependent patients. More than 3,000 scientific publications mention DSF in their title, and most of them are related to alcohol consumption.

After ingestion, alcohol (ethanol) is degraded in two steps:

$$\begin{array}{l} \;ADH\;ALDH\\ \text{Ethanol }(\text{CH3}.\text{CH2}.\text{OH})\to \text{Acetaldehyde }(\text{CH3}.\text{CHO})\to \\ \text{Acetate }(\text{CH3}.\text{COO}-) \end{array}$$

The first reaction is catalyzed by alcohol dehydrogenase (ADH), the second one by aldehyde dehydrogenase (ALDH). The final product, acetate, has no toxicity. By contrast, acetaldehyde (AcH), also known as ethanal, is much more toxic than ethanol. Ethanal is quite volatile, and at low concentration gives off a pleasant smell of green apple, whereas at higher concentrations, its smell becomes pungent. Acute AcH toxicity may involve in particular the nervous system (5). In long term exposure, AcH is also a carcinogen (6). Note that ALDH is only weakly expressed in 30–40% of Asian, individuals, preventing them from properly eliminating alcohol, which explains why many of them have a low tolerance to alcohol.

The potent DSF-induced ALDH inhibition is copper-dependent (7). *In vivo*, DSF is cleaved, giving rise to diethyldithiocarbamate, an efficient copper chelator (8). Through this mechanism, DSF inhibits copper-dependent enzymes, such as ALDH, abundant in the liver (9), or dopamine β-hydroxylase in the brain (10). The best described effect of DSF, but not the only one, is its toxicity in the presence of alcohol, and sometimes even in its absence, as discussed below.

There are two main places in the organism where the enzymes ADH and ALDH allow the degradation of alcohol to acetate. The first is the ALDH-rich liver, which plays a key role after alcohol drinking. The second, which is seldom mentioned but nevertheless quite important, is the microbiota of the digestive tract, with its billions of bacteria and fungi particularly abundant in the mouth and the large intestine. In some bacteria, the ADH enzymatic activity is significantly stronger than the ALDH one. As a result, in the presence of alcohol, such bacteria, including the commensal ones, trigger an increase in the concentration of toxic AcH (11). This might contribute to a higher frequency of mouth and throat cancers in alcohol-dependent patients (12).

In addition, some anaerobic bacteria and yeasts are able to convert glucose into ethanol (this “alcoholic fermentation” is the basis for the manufacturing of alcoholic beverages). Under certain culture conditions, it is possible, when supplying some of these microorganisms only with glucose, to generate alcohol and then AcH. Thus, the yeast *Candida albicans* is capable of producing high levels of toxic AcH, after glucose fermentation (11). It can thus be predicted that the toxicity of DSF should be particularly marked in people suffering from candidiasis.

Finally, other bacterial families, such as *Lactobacillus*, have an ALDH activity larger than that of ADH, which makes them good detoxifiers, by preventing the accumulation of AcH (6).

### Other Modes of Action of DSF

Although the DSF toxic effects occurring in the treatment of alcohol-dependent patients have been known for a long time, additional effects have been described more recently. It has been shown in particular that, *in vitro*, DSF can be cytotoxic for cancer cells (13). These results prompted the launch of three clinical trials including DSF in the treatment in prostate, pancreas and glioblastoma cancers (https://www.cancer.gov/about-cancer/treatment/clinical-trials/intervention/disulfiram). None of these trials, started in 2016 and 2017, has yet given rise to publication.

It was initially thought that these newly discovered effects were also due to the inhibition of ALDH, but this is not always true, and several other DSF targets have been identified. Thus, the protein NLP4, which is necessary for the cellular response to various stresses, is inhibited by DSF-copper complexes (13, 14). In addition, DSF can block an intracellular detoxifying pathway. It can inhibit the proteasome (15), a multi-protein complex required for the elimination of improperly folded proteins. DSF can also block the activation of NF-κB (15, 16), a key molecule in inflammatory stresses, known for inhibiting apoptosis.

*In vitro*, DSF can neutralize a DNA methyltransferase involved in DNA repair (17). It may also inhibit P-gp, a multidrug pump responsible for the extrusion of toxic molecules, which contributes to cellular resistance to many cytotoxic molecules (18). The effect of DSF on NF-kB, DNA repair, and P-gp may all contribute to the *in vitro* effects of DSF against tumor cell lines. The effect of DSF on P-gp has been more particularly studied in fungi and yeasts, offering a possible explanation for the antifungal effect of DSF (19). However, some authors have attributed DSF anti-fungal properties to its capacity to elicit oxidative stress in yeasts (20). Still *in vitro*, DSF also displayed toxic effect against *Plasmodium falciparum*, the causative agent of malaria (21), and also against some bacteria (22).

As mentioned previously, DSF can act as a copper chelator, thus inhibiting copper-dependent enzymes. Some bacteria express such enzymes, rendering them sensitive to DSF. However, it is unclear whether the anti-bacterial effect of DSF is due to copper depletion or to direct effects of copper complexation inside bacteria (8, 23).

Most demonstrations of an antibacterial effect of DSF were performed *in vitro* (24, 25), at concentrations not always compatible with its *in vivo* use. For instance, one study claims that DSF is toxic to *Mycobacterium tuberculosis*, including the dormant form, both *in vitro* and *in vivo* (24). In fact, the experimental protocol allowed the evaluation of the effect of DSF on the global bacterial load, but showed nothing on *in vivo* bacterial dormancy. In this study, the efficient dose of DSF would have been equivalent to 1,100 mg of DSF / day for a human of 70 kg, well above the dose tolerated by Lyme Disease patients (see below). Thus, the conclusions of this study still remain to be validated.

In summary, DSF is a pleiotropic drug with multiple targets, without specificity for one molecule or a single pathogen. Most of the reported anti-bacterial effects of DSF have been obtained *in vitro*, making it difficult to extrapolate for its *in vivo* use, especially when used in combination with antibiotics.

### A Clinical Trial With DSF for Treating Lyme Disease

In March 2019, Pr. Brian Fallon started a clinical trial using DSF and including 24 Lyme Disease patients (https://clinicaltrials.gov/ct2/show/NCT03891667). The results of Professor Fallon's study should provide important information in the near future. On the clinicaltrials.gov website, the Study Description indicates that DSF is active against Borrelia's dormant form. However, evidence to support this claim is not provided. The clinical trial document refers to three previous articles (22, 26, 27). In 2016, Pothineni et al. published an *in vitro* high-throughput screening of more than 4,300 drug candidates, against *Borrelia burgdorferi* grown to its stationary-phase (26). DSF appeared to be a very efficient bactericidal molecule for *Borrelia in vitro*, but no *in vivo* results have been reported yet. In 2017, Dr. Long has shown that, *in vitro*, DSF is cytostatic for Gram-positive bacteria, such as *Staphylococcus* or *Streptococcus*, but not for Gram-negative species (22). Finally, in 2019, Dr. Liegner reported three cases of patients who had been treated with DSF after a Lyme Disease that had lasted for several years with heavy treatments (27). For instance, at one point, one patient simultaneously took amoxicillin, clarithromycin, hydroxychloroquine, metronidazole, atovaquone / proguanil, and amitriptyline. After 9 years of illness he took DSF for 3 months: the symptoms of the Lyme Disease seem to have disappeared but the patient had a temporary psychiatric hospitalization. The second patient was on DSF for 6 weeks. The symptoms of the Lyme Disease improved but the treatment was stopped following a syncope, which resulted in a concussion and required hospitalization. In summary, the Liegner study reports three cases in which DSF seems to have been effective against late Lyme Disease, but in two of them neurological problems occurred during the treatment. These three cases have attracted considerable attention and raised great hopes in the Lyme Disease patient communities. However, in a recent talk at the 2019 ILADS Symposium, Dr. Liegner presented data on 30 Lyme Disease patients that he had treated with DSF. In 18 of them, DSF provoked either peripheral neuropathies or psychiatric problems, or both.

### DSF Neuronal Toxicity ?

For tens of years, it has been known that DSF can cause occasional and sometimes severe neuropathies (28). In optic neuropathies, with a partial loss of vision, recovery took about 6 months after stopping DSF (29). When DSF is used to treat alcohol dependence, the incidence of undesirable neuropathies has been estimated as 1/15,000 (30). As for the totality of the undesirable effects caused by the DSF, their frequency has been evaluated at 1 per 200–2,000 patients (9).

Are DSF associated neurological disorders (neuropathies or psychiatric problems) related to DSF anti-ALDH activity leading to AcH synthesis? It has been demonstrated that, *in vitro*, AcH can have an acute toxicity on neurons due to an increase of reactive oxygen species, but this observation has not been extended *in vivo* (5).

An AcH increased toxicity could theoretically occur even in the absence of alcohol intake, for example in patients with *Candida* infections, or harboring a high load of microorganisms capable of alcoholic fermentation. Alcoholic fermentation, typically performed by yeasts, should be distinguished from lactic fermentation, more common in anaerobic bacteria. A few cases have been reported of people suffering from Gut Fermentation Syndrome (31, 32). Such patients had up to 2 g/L of alcohol in their blood, without any alcohol intake. This alcohol was produced by fermentation by large colonies of the yeast *Saccharomyces cerevisiae* in their intestine.

Another DSF target is dopamine β-hydroxylase, a copper-dependent enzyme, responsible for converting dopamine (DA) to norepinephrine (NE) in noradrenergic neurons. This enzyme is mostly expressed in the brain, adrenal gland and liver (https://www.proteinatlas.org/ENSG00000123454-DBH/tissue). By inhibiting dopamine β-hydroxylase, DSF simultaneously reduces NE and elevates DA in these tissues. A link has been established between psychosis and DSF-induced increase of DA in the mesolimbic system (10, 33). Dopamine β-hydroxylase is also expressed in some peripheral sensory neurons and it has been suggested that neurotoxic products of catecholamines metabolism in nociceptors can cause neuronal dysfunction underlying neuropathic pain (34).

### Patient-Reported Outcomes

We have recently received from French associations of Lyme patients the results of an enquiry sent to their members suffering from persistent Lyme Disease. The main questions were: have you taken DSF as a treatment for your disease? Which benefits or side effects did you experience? 16 patients have answered.

The clinical features most frequently reported were major fatigue, articular pain and cognition complaints mainly involving memory, whether or not patients were seropositive for *Borrelia*. The results are presented in Table 1. The conclusions are: 13 out of 16 patients experienced DSF-induced toxic or side effects, mainly concerning the nervous system (neuropathies, headaches, dizziness, difficulty of concentration and expression, sleep disturbance, general pain increase, increase in general fatigue). Several patients reported a more specific increase in their osteo-articular pains, nausea or intestinal disorders.

When taking DSF, some patients simultaneously experienced both negative effects on some symptoms and improvement of others. All in all, 7 out of 16 patients perceived benefits mainly on fatigue and pain, especially after stopping DSF. Others could not differentiate whether partial improvements were due to DSF or to the antibiotics taken during the same period.

Some of DSF toxic effects observed in Lyme patients could be due in part to high initial DSF doses, similar to those used for alcohol-dependent patients. On the other hand, some of these effects could have been due to Jarisch Herxheimer reactions triggered by DSF-induced death of *Borrelia*. However, some patients, who had already experienced Jarisch Herxheimer reactions before, reported that some of the reactions encountered with DSF treatment were clearly of a different nature. Collectively, these observations suggest that patients with persistent Lyme Disease are more sensitive to the toxicity of DSF than people who have been treated for alcohol dependence, and that in these patients, DSF-induced toxicities are not all related to Jarish Herxeimer reactions.

## Discussion

Published scientific articles allow us to draw the conclusion that, *in vitro*, DSF can undoubtedly kill certain bacteria strains, and that *in vivo*, DSF can be toxic to both bacteria and the human body. These toxicities can be both acute and long-term.

One can propose different hypotheses to explain these toxicities. They might be mediated by the inhibition of copper-dependent enzymes, such as ALDH or dopamine β-hydroxylase, or the blocking of the NLP4 molecule, or through an oxidative molecule increase, and possibly through yet unidentified mechanisms. Part of these toxicities may also depend on the microbiota, in which some bacterial or yeasts species have a propensity to produce fermentation-derived toxic AcH. It would be worth testing if any intake of bacteria such as *Lactobacillus*, which have a high ALDH activity, could be used to counter the DSF-induced toxicities.

On the other hand, many studies have reported that patients with PTLDS have an increased sensitivity to pain, which can affect vision, hearing, touch, and even smell, as reviewed by Batheja et al. (35). These chronic pains can be related multiple to chemical sensitivity and chronic fatigue syndrome, in which the pain sensitivity is modified as well, as reported in Gulf war veterans (36). There is increasing evidence for abnormal sensory processing in these syndromes, with a low “unpleasantness threshold” for multiple types of sensory stimuli (37).

The differences observed for effective concentrations of DSF between alcohol-dependent patients and those suffering from PTDLS or SPPT could also be linked to a such central sensitization often observed in patients suffering from borreliosis (35).

It is necessary to understand why DSF toxicity appears particularly severe and frequent in patients with Lyme Disease, and to rapidly explore the reasons for such DSF toxicity in Lyme Disease animal models. Until we have the first answers to this question, it would be premature to consider DSF as the new miracle molecule for patients suffering from late disseminated Lyme Disease.

### Basic Science vs. Social Networks

Case reports are a very useful approach for drawing attention to the possible effectiveness of a new treatment. Undoubtedly, the case report published by Liegner (27) has played such a role. However, the next logical step should have been to examine the potential toxicity of DSF for Lyme patients. This could have been achieved first by using animal models, and then within a standardized clinical trial. These steps were rapidly short-circuited, due to the strong social demand for Lyme Disease treatments. This pressure is exerted largely by social networks, emphasizing their speed and efficiency, but at the same time a lack of analysis and scientific rigor.

### Importance of Patient-Reported Outcomes

Following the rapid spread of the idea that DSF could be a major improvement for the treatment of late Lyme Disease, hundreds of patients began using DSF in the hope of treating their disease. At this point, it is important to require, as we do here, on rapid feedback from the patients themselves. No one knows better than patients the severity and importance of secondary toxicities from treatment. They know themselves better than physicians, who sometimes tend to overestimate the benefit/risk ratios (38, 39).

The limitation of the present study is linked to the small number of included patients. This highlights the need for follow-up studies with a larger number of patients to specify the risk/benefit of DSF in late Lyme Disease. The results and experiences reported by the patients should be included in these studies to determine how many of them have truly benefited from DSF treatment. Aiming at distinguishing Jarish Herxheimer reactions due to bacterial die-off and toxic side effects of the drug will be an important issue. More generally, a patient survey will have to be designed to evaluate how many patients have benefited of DSF and how many have not. A long term follow up of the DSF treated patients using an online patient feedback tool will be necessary to determine if they have any relapse or stable remission. All this information is necessary to determine the risk/benefit ratio of DSF for Lyme Disease. This will require a close collaboration between patients, doctors and researchers.

## Data Availability Statement

All datasets generated for this study are included in the article/supplementary material.

## Ethics Statement

An ethical review process was not required according to national legislation and institutional requirements for the present study. Patient treatment was decided by their physician independently of the study. Patients associations were asked by the authors to collect patient-reported outcomes, which have been transferred anonymously to the authors. All patients that participated to this enquiry were volunteers and have given a written agreement to participate in this study.

21 patients had initially sent a patient-reported outcome. Following your recommendation, they were all asked to give their agreement to participate to this study. No one refused, but five of them did not answer. Therefore, the revised manuscript only presents the 16 cases of patients who sent their written consent.

## Author's Note

AT and HG are members of the scientific council of *Fédération Française contre les Maladies Vectorielles à Tiques* (FFMVT), and RG is president of FFMVT.

## Author Contributions

AT: analysis of the literature. HG, RG, and AT: synthesis and analysis of the patient-reported outcomes. RG: medical advice. AT and HG: writing the manuscript.

## Conflict of Interest

The authors declare that the research was conducted in the absence of any commercial or financial relationships that could be construed as a potential conflict of interest.
